# Expression of active caspase 3 in the rat lens after in vivo exposure to subthreshold dose of UVR-B

**DOI:** 10.1186/s12886-024-03315-x

**Published:** 2024-01-22

**Authors:** Konstantin Galichanin, Zhaohua Yu

**Affiliations:** Gullstrand lab, Section of Ophthalmology, Department of Surgical sciences, Uppsala University, Uppsala, SE-751 85 Sweden

**Keywords:** Lens, UVR-B, Active caspase 3, Time evolution, Cataract, In vivo, Western blot

## Abstract

**Purposes:**

The aim of this study is to investigate the time evolution of active caspase 3 within first 120 h in the rat lens after in vivo exposure to subthreshold dose of UVR-B.

**Methods:**

Twenty three six-week-old female albino Sprague-Dawley rats were exposed to subthreshold dose (1 kJ/m^2^) of UVR-B unilaterally and sacrificed at 24, 41, 70 and 120 h after exposure. Lenses were enucleated and active caspase 3 was detected by Western Blot. The time evolution of active caspase 3 was then plotted as a function of relative mean difference in active caspase 3 between exposed and nonexposed lenses.

**Results:**

There is expression of active caspase 3 in both exposed and nonexposed lenses but there is no difference in relative mean difference in active caspase 3 between exposed and nonexposed lenses in all four postexposure groups.

**Conclusions:**

Exposure to subthreshold dose of UVR-B does not induce apoptosis in the rat lens in vivo within first 120 h though there is a non-significant increase of active caspase 3 at 120 h. Increase in sample size might reduce the variation level in expression of active caspase 3 in the rat lenses.

## Introduction

The aim of this study is to investigate the time evolution of active caspase 3 during the first 120 h in the rat lens after in vivo exposure to subthreshold dose of UVR-B.

Apoptosis is the process of programmed death that involves cells [1]. It was found that caspase 3 is one of the main mediators in apoptosis. It acts as executioner protease and cleaves ICAD to release CAD [2]. CAD then degrades chromosomal DNA within the nuclei and causes chromatin condensation. In this article it is discussed how active caspase 3 is expressed in the rat lenses after in vivo exposure to subthreshold dose of UVR-B. Western blot is used as the method for investigation of expression of active caspase 3.

Ultraviolet radiation B (UVR-B) is the major risk factor for development of cortical cataract in humans. Sun is the main natural source of UVR-B. It is irradiated with 47 kJ/m^2^ in July and 2 kJ/m^2^ in December daily [3]. Ultraviolet radiation B causes direct DNA damage by producing pyrimidine dimers. Pyrimidine dimers are repaired by nucleotide excision repair. If pyrimidine dimers do not repair, they lead to apoptosis or mutations.

Previous research found that there is a mRNA expression of caspase 3 at 120 h in the rat lens after in vivo exposure to double threshold dose of 8 kJ/m^2^ [4] and subthreshold dose of 1 kJ/m^2^ [5] of UVR-B. It was also learned that there is no difference in relative mean difference in protein expression of active caspase 3 between exposed and nonexposed rat lenses within first 24 h (Manuscript submitted). Further, Michael et al. found that apoptosis is induced in the rat lens after in vivo exposure to UVR-B at the dose of 5 kJ/m^2^ [6]. In this study we investigated the time evolution of active caspase 3 in the rat lens after in vivo exposure to subthreshold dose of UVR-B within the first 120 h.

So, the purpose of the study is to investigate the time evolution of active caspase 3 within first 120 h in the rat lens after in vivo exposure to subthreshold dose of UVR-B.

## Materials and methods

### Animals

The experimental animal was the six-week-old albino Sprague-Dawley (SD) female rat (Taconic, Denmark). All animals were treated in accordance with the ARVO Statement for the Use of Animals in Ophthalmic and Visual Research. Ethical approval was obtained from the Uppsala Ethics Committee on Animal Experiments, protocol number 5.2.18–8927/16.

### Exposure to ultraviolet radiation

#### UVR source

**Fig. 1:**
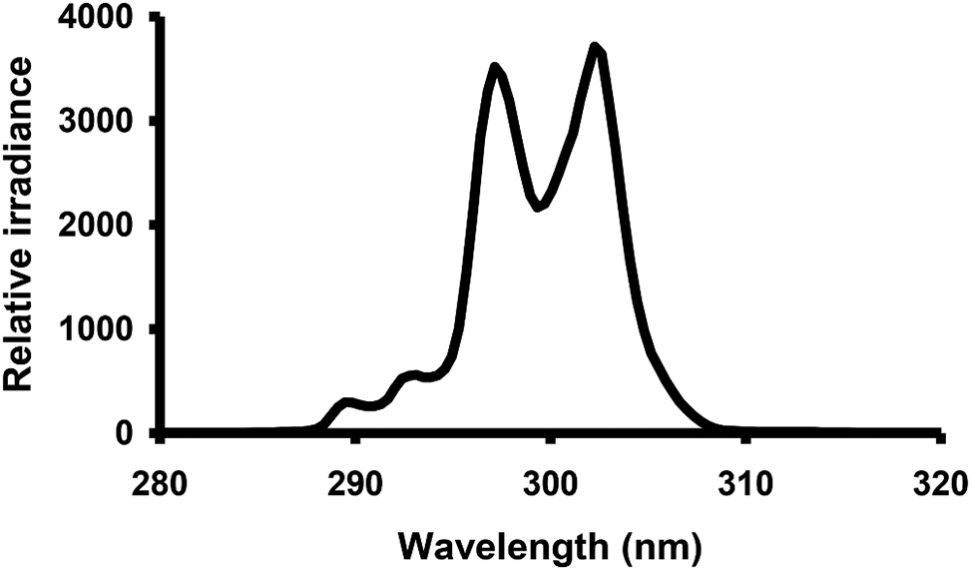
Spectral irradiance of ultraviolet radiation source

The high-pressure mercury arc lamp (model 6828; Oriel, Stratford CT) generated UVR-B at 300 nm (UVR-300 nm). The radiation was collimated, passed through a water filter, and focused on the entrance slit of a double monochromator. The emerging radiation had a spectral distribution centered at 300 nm with dual peaks at 297.5 and 302.6 nm with 10.2 nm full width at half maximum (Fig. 1) [7]. The thermopile (model 7101; Oriel, Stratford CT) calibrated to a US National Institute of Standard traceable source measured the intensity of UVR.

#### UVR exposure

A mixture of 90 mg/kg ketamine and 10 mg/kg xylazine was injected intraperitoneally to anesthetize the animal fifteen minutes before the exposure. Thereafter, the animal was placed in a rat holder [8], and tropicamide 10 mg/ml was instilled in both eyes to induce mydriasis. A subthreshold dose of 1 kJ/m^2^ of UVR-300 nm during 15 min [9] was applied to one eye of each animal, while the contralateral eye was shielded during the exposure [10].

After a pre-determined latency period, the rat was sacrificed by carbon dioxide asphyxiation. The eyes were enucleated, and the lenses were extracted. Remnants of the ciliary body were removed from the lens equator under a microscope, keeping the lens in balanced salt solution (BSS; Alcon, USA).

### Western blot

The lens samples were separated on a Mini-Protean TGX Stain-Free (12% gel) (Bio-Rad, Hercules, CA, USA), transferred onto nitrocellulose membrane (Bio-Rad, Hercules, CA, USA), and incubated sequentially with primary and appropriate horseradish peroxidase conjugated secondary antibodies. Primary antibodies were rabbit polyclonal cleaved caspase-3 antibody Asp175 (9661; Cell Signalling Technology, Inc., Danvers, MA, USA) for caspase 3 protein and mouse monoclonal anti-β-actin antibody (ab8227; Cambridge, UK) for β-actin protein that served as a reference protein. Secondary antibody was polyclonal peroxidase goat anti-rabbit HRP-linked IgG (7074 S; Cell Signalling technology, Inc., Danvers, MA, USA). Signals were detected using the Clarity Western ECL reagent kit (Bio-Rad, Hercules, CA, USA). Pictures were obtained by the imaging system Li-Cor Odyssey FC (LI-COR Biosciences, Lincoln, NE, USA) and analyzed with ImageJ software.

### Experimental design

Altogether 23 rats were used in the experiment. One eye in each animal was exposed in vivo to UVR-300 nm. Animals were sacrificed at 24, 40, 71 and 120 h after exposure to UVR-B. Samples from all lenses were processed for Western Blot of active caspase 3 protein. β-actin was used as a reference protein for each lens sample. Firstly, ratios between number of pixels of active caspase 3 and β-actin were calculated. Secondly, ratios between exposed and nonexposed lenses were found. Finally, relative mean difference in active caspase 3 between exposed and nonexposed lenses was calculated.

### Statistical parameters

The significance level and the confidence coefficient were set to 0.05 and 0.95, respectively, considering sample size.

## Results

**Fig. 2:**
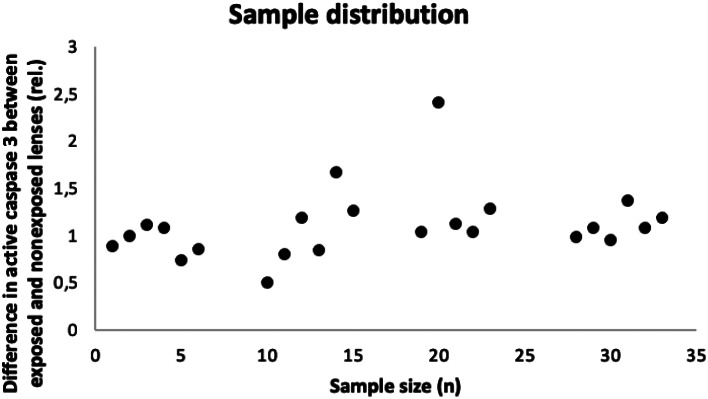
Sample distribution

Samples are normally distributed (Fig. 2).

**Fig. 3:**
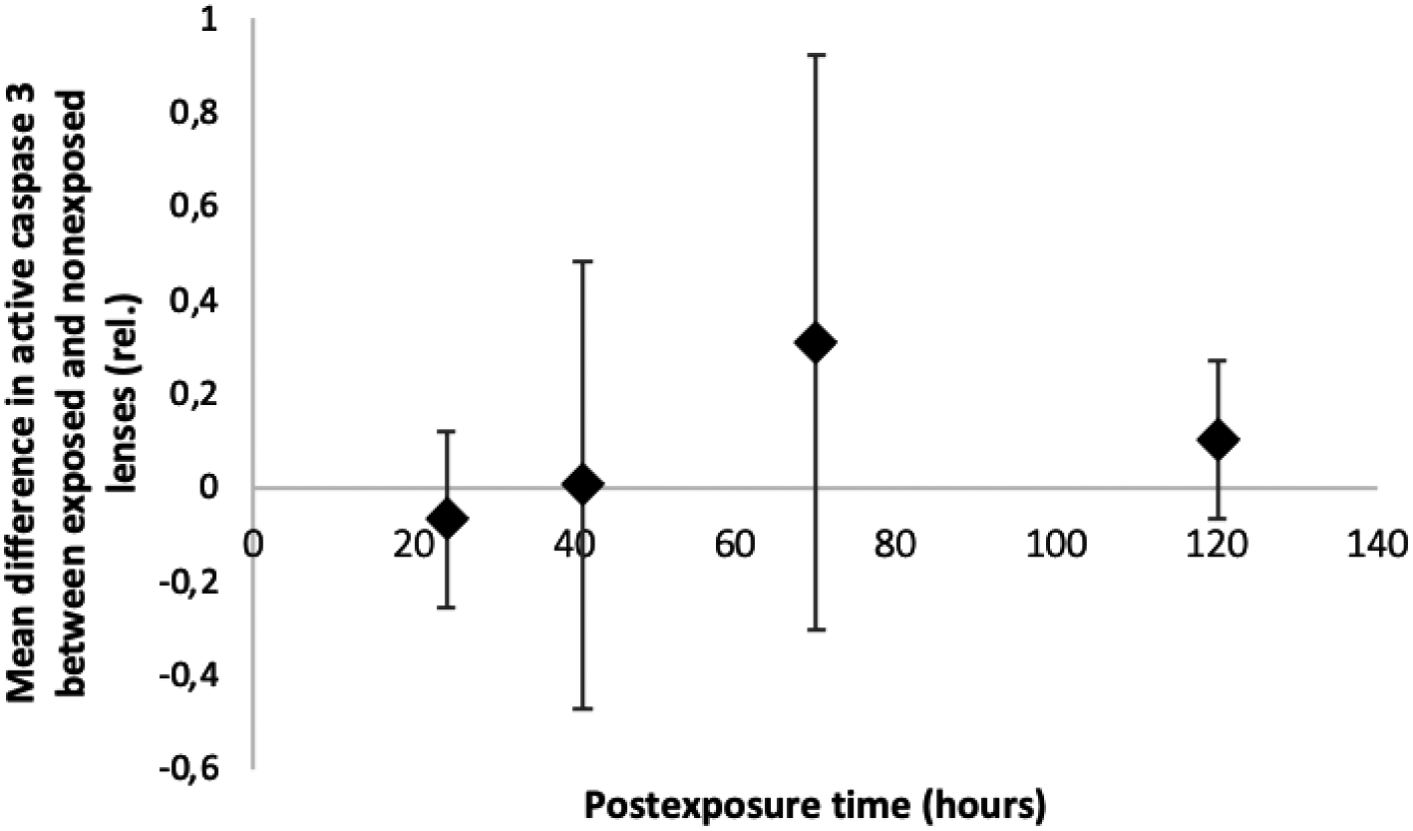
Evolution of mean difference in active caspase 3 in the rat lens after in vivo exposure to 1 kJ/m^2^ of UVR-B. Error bars are 95% confidence intervals for mean difference between exposed and nonexposed lenses

There is an expression of active caspase 3 for both exposed and nonexposed lenses in all four postexposure groups. It was found that there was no difference in relative mean difference in active caspase 3 between exposed and nonexposed lenses in all four postexposure groups (Fig. 3).

## Discussion

The study was designed to investigate the time evolution of active caspase 3 during the first 120 h in the rat lens after in vivo exposure to subthreshold dose of UVR-B.

The dose 1 kJ/m^2^ of UVR at 300 nm was selected to be a subthreshold dose [11] that induces expression of active caspase 3 in the rat lens in vivo [12, 13].

The time intervals of 24, 41, 70 and 120 h after the exposure were chosen based on previous investigation of Galichanin et al. (Manuscript submitted) that finds no difference in relative mean difference of active caspase 3 between exposed and nonexposed lenses within first 24 h postexposure.

The current finding that there is an expression of active caspase 3 for both exposed and nonexposed lenses in all four postexposure groups is in concordance with the results of Galichanin K. et al. (Manuscript submitted) that finds an expression of active caspase 3 in both exposed and nonexposed lenses within first 24 h postexposure. The probable explanation for this might be the normal protein turnover in the rat lens as discussed in the article of Galichanin K. et al. (Manuscript submitted).

Our observation that there is no relative mean difference in active caspase 3 between exposed and nonexposed lenses in all four postexposure intervals suggests that apoptosis does not occur within first 120 h followed by the exposure to subthreshold dose of UVR-B though there is a relatively narrow 95% confidence interval in 120 h postexposure group. If one increases the sample size in the postexposure groups, the relative mean difference between exposed and nonexposed lenses might become significant in 120 h postexposure group and turn to be increased in expression of active caspase 3. This might suggest that UVR-B exposure in the rat lens conforms the photochemical reaction of UVR-B to the lens followed by the apoptosis that occurs at later time intervals after in vivo exposure to subthreshold dose of UVR-B.

Biology of ultraviolet radiation cataract is in concordance with photochemical reaction of UVR-B to the lens. UVR-B induces apoptosis in the lens epithelial cells [6] followed by the osmodisregulation in the lens cortical cells [14]. It is supported by our previous studies where it was found that there is a time delay of 7 days in increased light scattering measurements [15] and of 5 days in increased mRNA expression of caspase 3 [4, 5] in the rat lens after in vivo exposure to UVR-B. One should take into consideration the postexposure time when design experiments on exposure of UVR-B to the lens.

In conclusion, exposure to subthreshold dose of UVR-B does not induce apoptosis in the rat lens in vivo within first 120 h. Increase in sample size might reduce the variation level in expression of active caspase 3 in the rat lenses after in vivo exposure to subthreshold dose of UVR-B specifically at 120 h postexposure.

## Data Availability

The datasets used and analysed during the current study are available from the corresponding author on reasonable request.
